# Accuracy and efficiency of automatic tooth segmentation in digital dental models using deep learning

**DOI:** 10.1038/s41598-022-13595-2

**Published:** 2022-06-08

**Authors:** Joon Im, Ju-Yeong Kim, Hyung-Seog Yu, Kee-Joon Lee, Sung-Hwan Choi, Ji-Hoi Kim, Hee-Kap Ahn, Jung-Yul Cha

**Affiliations:** 1 BK21 FOUR Project, Department of Orthodontics, Institute of Craniofacial Deformity, Yonsei University College of Dentistry, 50-1 Yonseiro, Seodaemun-gu, Seoul, 03722 Korea; 2 Research and Development Team, Laon Medi Inc., Sungnam, Korea; 3 Department of Computer Science and Engineering, Pohang University of Science and Technology, Pohang, Korea

**Keywords:** Orthodontics, Software

## Abstract

This study evaluates the accuracy and efficiency of automatic tooth segmentation in digital dental models using deep learning. We developed a dynamic graph convolutional neural network (DGCNN)-based algorithm for automatic tooth segmentation and classification using 516 digital dental models. We segmented 30 digital dental models using three methods for comparison: (1) automatic tooth segmentation (AS) using the DGCNN-based algorithm from LaonSetup software, (2) landmark-based tooth segmentation (LS) using OrthoAnalyzer software, and (3) tooth designation and segmentation (DS) using Autolign software. We evaluated the segmentation success rate, mesiodistal (MD) width, clinical crown height (CCH), and segmentation time. For the AS, LS, and DS, the tooth segmentation success rates were 97.26%, 97.14%, and 87.86%, respectively (*p* < 0.001, post-hoc; AS, LS > DS), the means of MD widths were 8.51, 8.28, and 8.63 mm, respectively (*p* < 0.001, post hoc; DS > AS > LS), the means of CCHs were 7.58, 7.65, and 7.52 mm, respectively (*p* < 0.001, post-hoc; LS > DS, AS), and the means of segmentation times were 57.73, 424.17, and 150.73 s, respectively (*p* < 0.001, post-hoc; AS < DS < LS). Automatic tooth segmentation of a digital dental model using deep learning showed high segmentation success rate, accuracy, and efficiency; thus, it can be used for orthodontic diagnosis and appliance fabrication.

## Introduction

The first step of successful orthodontic treatment is accurate diagnosis and establishing an optimised treatment plan. An orthodontist collects various types of patient data, such as clinical examinations, radiographs, intra- and extra-oral clinical photographs, and dental models, prepares a problem list, sets treatment goals, and establishes orthodontic treatment plans accordingly. Several treatment options are typically available depending on patients’ individual needs, even with similar orthodontic conditions. In this situation, a diagnostic model setup showing expected treatment outcomes helps patients and orthodontists make decisions.

Manual setup using plaster models requires considerable time and effort in an orthodontic lab. Furthermore, comparing the various options using plaster models is difficult and cannot be used for all treatment cases. In contrast, a virtual setup^1^ with a digital model provides information on changes in tooth movement, angle, and arch form by superimposition, which helps determine various treatment options. Moreover, studies have found no significant difference in reliability when compared with a manual setup^2^. At present, digital orthodontic solutions, such as computer-aided-design/computer-aided manufacturing (CAD/CAM) software and intra- and extra-oral scanners, are used by orthodontists to diagnose and fabricate orthodontic appliances^3,4^.

Diagnostic setup aids in extraction, interproximal reduction, anchorage preparation, and treatment mechanics, enabling precise treatment plans^5,6^. Hou et al.^7^ reported that using a digital setup changed treatment plans in 23.6% of diagnostic cases, and the reliability of the treatment plan selected was enhanced, especially in difficult cases and cases in which residents made the decision with little clinical experience. Many orthodontists routinely use virtual setups for various digital orthodontic procedures, such as clear aligners^8^, individual jigs for indirect bonding^9^, custom brackets, and arch wires^10^.

Representative commercial orthodontic CAD/CAM software, such as OrthoAnalyzer (3Shape, Denmark), SureSmile (Dentsply Sirona, USA), OrthoStudio (Maestro 3D, Italy), OrthoCAD, (Align Technology, USA) ClinCheck, and Autolign (Diorco, Korea), contain functions for segmentation of digital tooth models, three-dimensional (3D) tooth movement, virtual setup, and orthodontic device production. The early versions of orthodontic CAD/CAM software were less efficient because they required considerable manual intervention. Since then, the algorithms have improved, increasing the accuracy and efficiency of tooth segmentation, classification, and automation levels.

Accurate tooth segmentation is essential for model setup. The tooth segmentation methods used in digital models may affect the model analysis and setup as the shape of the proximal surface and mesiodistal (MD) width recorded may vary depending on the method. The MD width of each tooth in a digital setup model was shown to be shorter than the conventional plaster setup model in most cases^11^, and the arch perimeters of the virtual setup of the digital model tended to be shorter than those of a manual setup with a plaster model^12^. This is because the plaster model can easily reproduce the proximal surface of the tooth by trimming after segmentation, whereas the digital model obtains a hollow image based on surface data; thus, reproducing the shape of the proximal surface that cannot be scanned is difficult.

Several studies have introduced their own methods for 3D object segmentation. Various deep learning techniques have been developed to segment 3D objects. General segmentation methods can be classified into region-based and feature curve-based methods^13^. Region-based methods include *k*-means clustering, random walk, fitting primitives, and fast marching watershed, which distinguish the mesh using the similarity of surrounding regions but have difficulties in defining tooth models with variable shapes as semantic regions. Feature curve-based methods include the snake evolution method, morphological skeleton extraction, and plan-view range image; among these, the snake evolution method is considered the most popular method for tooth segmentation^14^. It is, however, sensitive to curvature noise and has poor reliability when the scanned data are poor^13^. The specificity of the tooth shape, the tight line between teeth and gingiva, and the close contact between adjacent teeth make automatic and precise tooth segmentation difficult^15^.

Recently, various deep learning techniques have been developed to segment 3D objects, and point cloud deep learning models^16–18^ that work directly with raw point clouds are active research subjects^19^. Dynamic graph convolutional neural networks (DGCNNs) have also been applied to segment 3D objects, which can be further used for the segmentation and classification of digital dental models^20^. DGCNNs improve segmentation performance by combining edge convolution based on PointNet^16^, a deep learning model using point clouds, which are the basic units for reproducing 3D objects. This technique can be utilized in tooth segmentation and classification.

The purpose of this study is to compare and evaluate the accuracy and efficiency of the DGCNN-based segmentation and classification of digital dental models with two existing commercially available software programs. Herein, ‘the three different tooth segmentation methods of the digital tooth model do not significantly differ in the success rate, time, and tooth size’ is set as the null hypothesis.

## Results

We analysed the reliability of measurements and found that the intraclass correlation coefficients (ICCs) for the intra-rater reliability were 0.987–0.997 (Table 1). In the final assessment, we reviewed the intra-rater agreement (success vs failure) as well as Cohen’s kappa statistic and its *p*-value. Table 2 shows the statistical analysis results of the degree of agreement within the evaluator on the success and failure of tooth segmentation with nominal variables. The range of kappa values in the three different segmentation types ranged from 0.885 to 1.000, showing very high reproducibility between the first and second assessments.

**Table 1:** Reliability analysis of tooth measurement.

Segmentation type	MD width		CCH	
	ICC (95% CI)	*p* value	ICC (95% CI)	*p* value
LS	0.994 (0.992–0.995)	< 0.0001	0.992 (0.990–0.994)	< 0.0001
DS	0.987 (0.983–0.989)	< 0.0001	0.989 (0.985–0.991)	< 0.0001
AS	0.997 (0.997–0.998)	< 0.0001	0.991 (0.998–0.993)	< 0.0001
REF	0.997 (0.997–0.998)	< 0.0001	0.993 (0.990–0.994)	< 0.0001

MD width and CCH, presented as continuous variables, were verified for intra-rater reliability using ICC. The ICC of MD width and CCH were 0.994–0.997 and 0.989–0.993, respectively, showing very high reproducibility.

ICC > 0.7: excellent.

**Table 2:** Kappa statistics of agreement of measurement results on success/failure of tooth segmentation.

Segmentation type			First			Kappa	*p* value
			Success	Failure	Total		
LS	Second	Success	271 (100.0%)	0 (0.0%)	271 (96.8%)	1.000	< 0.0001
		Failure	0 (0.0%)	9 (100.0%)	9 (3.2%)		
		Total	271 (100.0%)	9 (100.0%)	280 (100.0%)		
DS	Second	Success	244 (99.6%)	0 (0.0%)	244 (87.1%)	0.984	< 0.0001
		Failure	1 (0.4%)	35 (100.0%)	36 (12.9%)		
		Total	245 (100.0%)	35 (100.0%)	280 (100.0%)		
AS	Second	Success	270 (99.3%)	0 (0.0%)	270 (96.4%)	0.885	< 0.0001
		Failure	2 (0.7%)	8 (100.0%)	10 (3.6%)		
		Total	272 (100.0%)	8 (100.0%)	280 (100.0%)		

Success and failure of segmentation, presented as nominal variables using Cohen's kappa, indicated very high evaluation reproducibility from 0.885 to 1.000.

Kappa values were interpreted as follows: poor,  < 0.0; slight, 0.0 to 0.2; fair, 0.2 to 0.4; moderate, 0.4 to 0.6; substantial, 0.6 to 0.8, and almost perfect, 0.8 to 1.0.

The success rates for automatic segmentation were 97.26% and 97.14% in the AS and LS, respectively, which were higher than the 87.86% success rate of the DS (Table 3). These rates were significantly different between the three groups (*p* < 0.001, post hoc test: LS, AS > DS).

**Table 3:** Comparison of success rate among three methods.

	LS	DS	AS	*p*^a^ value	Post hoc test
Success	816 (97.14%)	738 (87.86%)	817 (97.26%)	< 0.001*	LS, AS > DS
Failure	24 (2.86%)	102 (12.14%)	23 (2.74%)		

Success rates for automatic segmentation were 97.26 and 97.14% for the AS and LS, respectively, which were higher than the 87.86% success rate of the DS.

Data are given as n (percentage).

^a^*p* values were derived from Cochran's Q test; *p < 0.05.

Furthermore, we compared the MD width, height, and segmentation time of the three segmentation types (Table 4). The means (95% CI) of the MD widths were 8.28 (8.15, 8.41), 8.63 (8.49, 8.76), and 8.51 (8.37, 8.65) mm in the LS, DS, and AS, respectively. There was a significant difference in the MD widths of the three groups (*p* < 0.001, post hoc test: DS > AS > LS). The CCH means (95% CI) were 7.65 (7.52, 7.78), 7.52 (7.39, 7.65), and 7.58 (7.45, 7.70) mm in the LS, DS, and AS, respectively. CCH was significantly higher in the LS than the DS and the AS (*p* < 0.001, post hoc test: LS > DS, AS). The means (95% CI) of segmentation times were 424.17 (404.28, 444.05), 150.73 (140.70, 160.77), and 57.73 (54.43, 61.04) s in the LS, DS, and AS, respectively. We found a significant difference in the segmentation times of the three groups (*p* < 0.001, post hoc test: LS > DS > AS).

**Table 4:** Comparison of MD width, CCH, and segmentation time among three segmentation groups.

	LS	DS	AS	REF	*p*^a^ value	Post hoc test
MD width (mm)	8.28 (8.15, 8.41)	8.63 (8.49, 8.76)	8.51 (8.37, 8.65)	8.52 (8.40, 8.63)	< 0.001*	DS > REF, AS > LS
CCH (mm)	7.65 (7.52, 7.78)	7.52 (7.39, 7.65)	7.58 (7.45, 7.70)	7.62 (7.50, 7.74)	< 0.001*	LS, REF > DS, AS
Time (sec)	424.17 (404.28, 444.05)	150.73 (140.70, 160.77)	57.73 (54.43, 61.04)		< 0.001*	LS > DS > AS

MD width and CCH showed statistically significant differences, depending on segmentation method. The segmentation time also showed statistically significant differences in the three groups, with the AS having the least manual intervention being the shortest.

Data are given as the mean (95% confidence interval).

^a^*p* values were derived from Friedman test; Shapiro–Wilk’s test was employed to test the normality assumption; *p < 0.05.

We compared the tooth size error by tooth group (Table 5). The means (95% CI) of the MD width error ranged from −0.31 (−0.35, −0.28) to −0.08 (−0.14, −0.02) mm, −0.09 (−0.14, −0.04) to 0.68 (0.60, 0.76) mm, and −0.35 (−0.39, −0.31) to 0.61 (0.51, 0.71) mm in the LS, DS, and AS groups, respectively. There were statistically significant differences in all tooth groups (*p* < 0.001, post hoc: DS > AS > LS in upper incisal, lower incisal, lower canine, and lower premolar; DS, AS > LS in upper canine, upper and lower molar, DS > LS, AS in upper premolar). The means (95% CI) of CCH error ranged from −0.03 (−0.07, 0.01) to 0.00 (−0.02, 0.02) mm, −0.21 (−0.26, −0.16) to −0.09 (−0.14, −0.04) mm, and −0.11 (−0.15, −0.07) to −0.06 (−0.08, −0.04) mm in the LS, DS, and AS groups, respectively. There were statistically significant differences in all tooth groups (*p* < 0.001, post hoc test: LS > DS, AS).

**Table 5:** Comparison of MD width and CCH among the three methods by tooth group.

Variable		MD width (mm)					CCH (mm)				
		LS	DS	AS	*p*^a^ value	Post-hoc test	LS	DS	AS	*p*^a^ value	Post-hoc test
Upper	Incisal	−0.26 (−0.29, −0.23)	0.07 (0.03, 0.11)	0.00 (−0.05, 0.05)	< 0.001	DS > AS > LS	−0.01 (−0.02, 0.00)	−0.21 (−0.26, −0.16)	−0.11 (−0.15, −0.07)	< 0.001	LS > AS, DS
	Canine	−0.21 (−0.26, −0.17)	−0.06 (−0.10, −0.01)	−0.15 (−0.21, −0.09)	< 0.001	DS, AS > LS	−0.02 (−0.04, −0.01)	−0.19 (−0.25, −0.13)	−0.10 (−0.12, −0.07)	< 0.001	LS > AS, DS
	Premolar	−0.31 (−0.35, −0.27)	−0.06 (−0.09, −0.02)	−0.35 (−0.39, −0.31)	< 0.001	DS > LS, AS	−0.03 (−0.06, −0.01)	−0.14 (−0.19, −0.08)	−0.09 (−0.12, −0.06)	< 0.001	LS > AS, DS
	Molar	−0.08 (−0.14, −0.02)	0.68 (0.60, 0.76)	0.61 (0.51, 0.71)	< 0.001	DS, AS > LS	−0.02 (−0.06, 0.02)	−0.11 (−0.18, −0.04)	−0.08 (−0.12, −0.05)	< 0.001	LS > AS, DS
Lower	Incisal	−0.26 (−0.30, −0.23)	−0.01 (−0.06, 0.04)	−0.07 (−0.11, −0.04)	< 0.001	DS > AS > LS	0.00 (−0.01, 0.01)	−0.18 (−0.23, −0.13)	−0.11 (−0.13, −0.08)	< 0.001	LS > AS, DS
	Canine	−0.26 (−0.32, −0.20)	0.06 (−0.02, 0.14)	−0.14 (−0.18, −0.10)	< 0.001	DS > AS > LS	0.00 (−0.02, 0.02)	−0.13 (−0.20, −0.07)	−0.09 (−0.13, −0.06)	< 0.001	LS > AS, DS
	Premolar	−0.31 (−0.35, −0.28)	−0.06 (−0.10, −0.03)	−0.15 (−0.19, −0.12)	< 0.001	DS > AS > LS	−0.03 (−0.07, 0.01)	−0.15 (−0.18, −0.11)	−0.10 (−0.12, −0.08)	< 0.001	LS > AS, DS
	Molar	−0.13 (−0.17, −0.09)	0.24 (0.19, 0.29)	0.20 (0.16, 0.24)	< 0.001	DS, AS > LS	0.00 (−0.01, 0.01)	−0.09 (−0.14, −0.04)	−0.06 (−0.08, −0.04)	< 0.001	LS > AS, DS

The means of the MD width error ranged from −0.31 to −0.08 mm, −0.09 to 0.68 mm, and −0.35 to 0.61) mm in the LS, DS, and AS groups, respectively. There were statistically significant differences in all tooth groups (*p* < 0.001, post hoc: DS > AS > LS in upper incisal, lower incisal, lower canine, and lower premolar; DS, AS > LS in upper canine, upper and lower molar, DS > LS, AS in upper premolar). The means of the CCH error ranged from −0.03 to 0.00 mm, −0.21 to −0.09 mm, and −0.11 to −0.06 mm in the LS, DS, and AS groups, respectively. There were statistically significant differences in all tooth groups (*p* < 0.001, post hoc test: LS > DS, AS).

Data are given as the mean (95% confidence interval).

^a^*p* values were derived from Friedman test; Shapiro–Wilk’s test was employed to test the normality assumption; *p < 0.05.

We observed significant effects of tooth group, software, MD width/CCH, and first-order interactions of Tooth Group * Software, Tooth Group * MD width/CCH, and Software * MD width/CCH on the tooth size errors in the generalised linear mixed model (GLMM) analysis (*p* < 0.05) (Table 6). Results showed that the tooth size errors were statistically different (*p* < 0.001) depending on the software used, and the post hoc test showed that DS (−0.019) > AS (−0.052) > LS (−0.121).

**Table 6:** Statistical analysis of main effects and first-order interactions affecting measurement error using GLMM.

Variable	DF	F	*p*^a^ value	Post hoc test^b^
Intercept^c^	1:4723	319.03	< 0.001	
Tooth group	3:4723	296.67	< 0.001	Molar (0.102) > incisal (−0.098), Canine (−0.110) > premolar (−0.149)
Software	2:4723	70.00	< 0.001	DS (−0.019) > AS (−0.052) > LS (−0.121)
MD width/CCH	1:4723	32.92	< 0.001	MD width (−0.043) > CCH (−0.084)
Tooth group * Software	6:4723	24.70	< 0.001	
Tooth group * MD width/CCH	3:4723	201.37	< 0.001	
Software * MD width/CCH	2:4723	428.81	< 0.001	

The tooth size errors were statistically different (*p* < 0.001) depending on the software used, and the post hoc test showed that DS (−0.019) > AS (−0.052) > LS (−0.121).

*DF* degrees of freedom.

*F* F value.

^a^*p* values were derived from a generalised linear mixed model.

^b^Category (estimated mean) was presented for Bonferroni’s corrected post hoc test.

^c^Intercept represents the mean value of the response variable when all predictor variables in the model are zero.

## Discussion

In orthodontics, artificial intelligence, including deep learning, can be applied to diagnosis and treatment planning for orthodontic extractions or orthognathic surgery^21–23^, automated cephalometric landmarking^24,25^, diagnosis of impaction^26^, determination of skeletal maturity for growth stage evaluation^27^, and automatic segmentation and setup of digital models^14,19,28^. Reducing the time and effort required for simple tasks for diagnosis and appliance fabrication allows the users to focus more on making decisions.

In this study, we verified the MD width and CCH, presented as continuous variables, for intra-rater reliability using ICC. The success and failure of segmentation, presented as nominal variables using Cohen's kappa, indicated very high evaluation reproducibility: ICC and Cohen’s kappa were 0.987–0.997 and 0.885–1.000, respectively. In addition, this study showed statistically significant differences in segmentation success rate, time, and size of segmented teeth using three different orthodontic CAD/CAM programs; thereby, rejecting the null hypothesis.

We designed the DGCNN-based segmentation model in two stages to prevent degradation of the segmentation performance due to differences in number of vertices between tooth and gingiva. In the first stage, the digital dental model was segmented into gingiva and dentition using the two-class DGCNN model. In the second stage, the digital dental model was segmented into individual tooth and gingiva using the seventeen-class DGCNN model after adjusting the number of gingiva vertices, which were segmented in the first stage, not to exceed twice the number of individual tooth vertices.

DGCNN using point clouds is advantageous for semantic segmentation and classification of a digital dental model, but it suffers from poor resolution for tooth margins. We attempted to obtain a clear tooth margin by supplementary use of curve-based mesh segmentation using skeleton/pruning algorithm. However, in some cases, the closed loop may not be formed due to the unclear curvature of the scanned data, or a closed loop may be formed in the wrong area, such as the tooth groove. Therefore, the segmentation and classification by DGCNN was optimized by supplementing the curvature-based mesh segmentation.

To compare and evaluate the accuracy and efficiency of the DGCNN-based segmentation model, we used two existing commercially available software. Both software packages used for comparison (i.e., OrthoAnalyzer and Autolign) were selected for the following reasons: the first reason is popularity; both are popular with orthodontists. Second, we considered their functionality for the tooth segmentation; OrthoAnalyzer is characterized by the need to set precise MD points for tooth segmentation, and Autolign has the need to set approximate MD points. Third, we considered their versatility. Some orthodontic software packages create closed working environments that prevent exportation of segmented teeth. OrthoAnalyzer and Autolign can export segmented teeth as stereolithography files, which can be imported to Meshmixer and Geomagic Control X software for success/failure determination and tooth-size measurement.

This study presented the digital dental model segmentation success and failure for its clinical applications. As the purpose of tooth segmentation is to diagnose and fabricate orthodontic appliances, such as custom brackets, clear aligners, and indirect bonds, accurate tooth surface models are essential to fabricate orthodontic appliances, and defects cannot be allowed. Considering the width and height of the bracket base and the undercut needed to obtain the retention of the removable application, the cervical ± 25% line was set as the success baseline. Therefore, criteria for determining whether the segmentation was successful or not include the cervical margin of the segmented tooth not deviating beyond ± 25% of the cervical margin of the actual tooth and finding no defects in the occlusal or incisal edge of the segmented tooth.

A high segmentation success rate increases user convenience by reducing the time and effort required to modify segmentation splines. This study showed high segmentation success rate (Table 3) in all three groups. However, the success rates of LS and AS (97.14% and 97.26%) were significantly higher than the success rate of DS (87.86%). These findings imply that there are differences in the success rates of different segmentation methods. In contrast to segmentation of general objects, tooth segmentation has to work on the complex intersection of concave regions (e.g. tooth-gingival margin, tooth groove, and interproximal area), for which traditional geometry-based segmentation is typically used. Various tooth segmentation algorithms have been introduced to overcome these limitations. However, region-based and feature curve methods still involve some limitations, such as difficulty obtaining high-quality segmentation results and reduced efficiency due to complex implementation procedures. The recent developments in deep learning require little manual intervention and have low algorithm complexity and high accuracy. In this study, the AS method based on DGCNNs exhibited satisfactory success rates.

The MD width of the experimental group used the results provided by each software program. Consequently, results varied depending on different measurement and calculation methods. OrthoAnalyzer, used in the LS, set the mesial and distal points of individual teeth before tooth segmentation and calculated the MD width using the virtual plane formed by the screen view at the MD point setting. In contrast, Autolign and LaonSetup used in the DS and the AS, respectively, were normalised after tooth segmentation to calculate the MD width. Even if the software user sets the MD points precisely on an unsegmented dental model, they are impossible to set in the occlusion area of the interproximal region. Owing to the location characteristics of the measurement points, the MD width measured before tooth segmentation is likely to be measured more conservatively than when measured on the segmented teeth. In this study, the MD width of LS also showed an error of −0.35 mm and −0.23 mm when compared with DS and AS, respectively.

The MD widths of upper molars were recorded as larger in DS and AS groups than in the LS group. This may be because of the characteristic shape of the upper molars and the method of MD width measurement. An upper molar often forms a parallelogram in occlusal view, in contrast to other teeth whose height of contour on the MD surface is clear. When an upper molar is in the shape of a parallelogram, the heights of contour in mesial and distal surfaces show many differences in the bucco-lingual position. Without considering these morphological features, the MD width of a normalised upper molar can cause errors in measurement, leading to inaccurately large sizes. Therefore, the MD width measurements of upper molars require corrections.

A limitation of digital models is that measuring accurate MD width is difficult because of the presence of occlusions in the interproximal area. When using a plaster model, the adjacent surface is reproduced naturally during the teeth section, but in the case of a digital model with only surface information, the occlusions in contact with the adjacent area during segmentation remain empty after segmentation. To solve this problem, Kim et al.^14^ proposed an image reconstruction method for an adjacent occlusion using a generative adversarial network and obtained an average improvement of 0.004 mm compared with the conventional method.

CCH can affect vertical bracket positioning and thus requires evaluation of accuracy. Compared with the DS and AS, LS had fewer errors in the whole tooth group, and DS and AS produced shorter CCHs than reference group (REF). However, according to the CCH of each tooth group (Table 5), the maximum average error was −0.21 mm, which is not likely to cause problems in diagnosis and appliance fabrication, and it is considered clinically acceptable.

This study performed repeated measurement of digital dental models conducted with three different segmentation methods, and there were within-subject correlations that were not independent. In addition, segmentation failure resulted in a missing value because the MD width and CCH could not be measured. Therefore, we used GLMM as a statistical method to analyse the main effects and the first-order interactions of the MD width and CCH errors. The segmentation success rate of the DS was lower than that of the remainder of the group, but the mean error according to the segmentation method was the lowest in the DS. This was the result of evaluating successful teeth segmentation and excluding missing values due to failure of segmentation. In addition, the mean error value results between groups of post hoc tests were within 0.12 mm and thus clinically acceptable.

The mean segmentation time of the AS was 57.73 s, which was shorter than those of the DS and the LS, with means of 150.73 and 424.17 s, respectively, presenting significant differences in efficiency. This was attributed to the differences in the segmentation processes; in all three experimental groups, the digital model was orientated to the coordinate system, but the subsequent process differed for each group. In the case of LS, which required precise marking of the mesial and distal points of all teeth, the segmentation time was the longest due to the necessity of axes specification of each tooth. Similarly, the DS also required marking of mesial and distal points for all teeth but did not require precise marking, so the time required for point designation was short. In the case of AS, segmentation and classification were performed without manual intervention after orientation; thus, it took the shortest time for segmentation. As the convenience of using segmentation increases and the requirement of manual intervention decreases, it is important to automate segmentation and reduce the time and effort required for correction by decreasing the segmentation failure rate.

A limitation of this study is that we used digital models of permanent dentition in good condition without teeth and gingival defects. Therefore, it is not possible to determine the tooth segmentation ability in cases such as a missing tooth, severe wearing, dental caries, partial eruption, and third molar. In subsequent studies, it will be necessary to use digital dental models of various conditions.

Because the OrthoAnalyzer software used in LS was used to obtain the reference data, bias may have occurred. To reduce the potential bias, the splines of all segmented teeth in the REF method were corrected. Moreover, the MD width of REF was measured using Geomagic Control X software. There was no correction after segmentation across experimental groups (i.e., LS, DS, and AS), and raw MD width data provided by software were used. Therefore, depending on the segmentation accuracy and the method of calculating MD width, values may differ from REF.

This study used different automatic tooth segmentation software in different groups. Because the three software programs were not developed and distributed simultaneously, their performances may vary depending on the software version. Moreover, the software continues to be updated to new versions; thus, improvements in accuracy, convenience, and speed of segmentation and classification can be expected. In addition, because the CAD/CAM software for orthodontics used in this study was distributed for commercial use, the detailed algorithms were not disclosed, making a direct comparison of the segmentation methods impossible. Therefore, this study focused on comparing the software from the point of view of a user and compared the use of the software and results of tooth segmentations.

## Materials and methods

### Digital model selection

This retrospective study was approved by Yonsei University Dental Hospital Institutional Review Board (IRB No. 2-2021-0033) and passed the exemption review of informed consent on the use of patients’ intraoral scan data. All clinical examinations were conducted in accordance with the Declaration of Helsinki.

Among the 1005 digital dental model sets of patients treated by the Department of Orthodontics at Yonsei University Dental Hospital between January 2010 and February 2019, we selected 546 digital dental models satisfying the following criteria.

The inclusion criteria of the digital models were as follows:

- Over 14-year-old orthodontic patients with second molars eruption.
- Digital dental models without any defects in the teeth and gingiva.
- Mild and moderate crowding.

The exclusion criteria of the digital models were as follows:

- Congenital tooth deformity.
- Severe dental caries and tooth wear.
- Congenital or acquired missing tooth.
- Supernumerary tooth.
- Severe crowding.

We used 516 dental models for deep learning-based tooth segmentation training, and the 30 dental models to evaluate the accuracy and efficiency of segmentation and classification of digital tooth models based on DGCNN model using two commercially available software programs.

### Deep learning process

**Figure 1:**
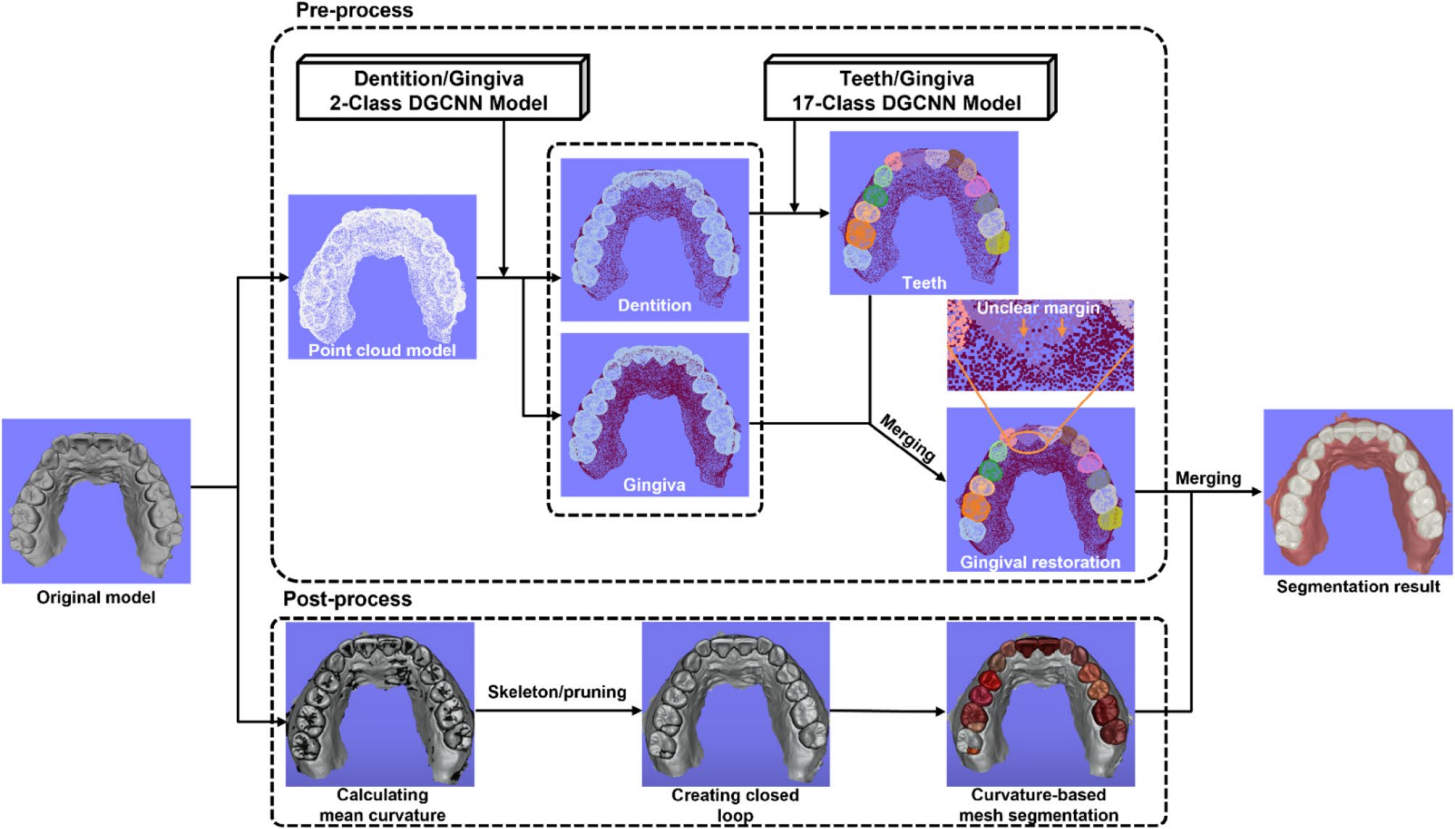
Schematic view of the deep learning process. After the original dental model was converted to a point cloud model, the dentition and the gingiva were segmented using the two-class DGCNN model. To increase the accuracy of semantic segmentation, we segmented individual teeth and gingiva using the seventeen-class DGCNN model with the vertices of the point cloud of the gingiva reduced to less than twice that of the tooth group. The gingival vertices were then restored. Since some of the segmentation results using DGCNN showed an unclear teeth margin, curvature-based mesh segmentation was used as post-processing to segment the teeth margin. The images of the digital dental model used in this figure were obtained using MeshLab (ver. 1.3.4 BETA, ISTI-CNR, Italy) and Unity Editor (ver.2020.3.23f1, Unity Technologies, USA) software.

We performed segmentation and classification of digital dental models based on DGCNN^20^ as shown in Fig. 1. We extracted vertices from the digital dental model and converted into a point cloud model. When performing the two-class DGCNN model method that segments dentition and gingiva, we performed uniform sampling so that the number of points classified as gingiva was approximately twice that of the dentition. In addition, the seventeen-class DGCNN model was implemented to segment and classify individual teeth and gingiva. The hyperparameters of the DGCNN model used in this study can be found as Supplementary Fig. S1 online.

As a post-processing process to determine precise tooth proximal and cervical margin, we divided the interdental area based on the mesh curvature^29^. We established the tooth margin, that showed a negative value as compared to the mean curvature of the tooth, as a feature vertex, and formed a closed loop by applying the skeleton pruning algorithm^30^. We obtained segmentation results with clear margins by merging the teeth and gingiva segmented by the DGCNN model and the teeth segmented by a mesh curvature closed loop.

### Sample size and power calculation

Thirty digital dental models were used in the experiment. As 28 segmented teeth were evaluated per digital dental model, 840 teeth were evaluated in each of the four methods. With an observed sample size of n = 30 per group, power analysis of variance (two-tailed) conducted a posteriori using G*Power software version 3.1.9.2 (Franz Faul, Universität Kiel, Kiel, Germany) indicated > 99% power was needed to detect a medium effect size (Cohen’s d = 0.25) at a significance level of 0.05.

### Tooth segmentation

**Figure 2:**
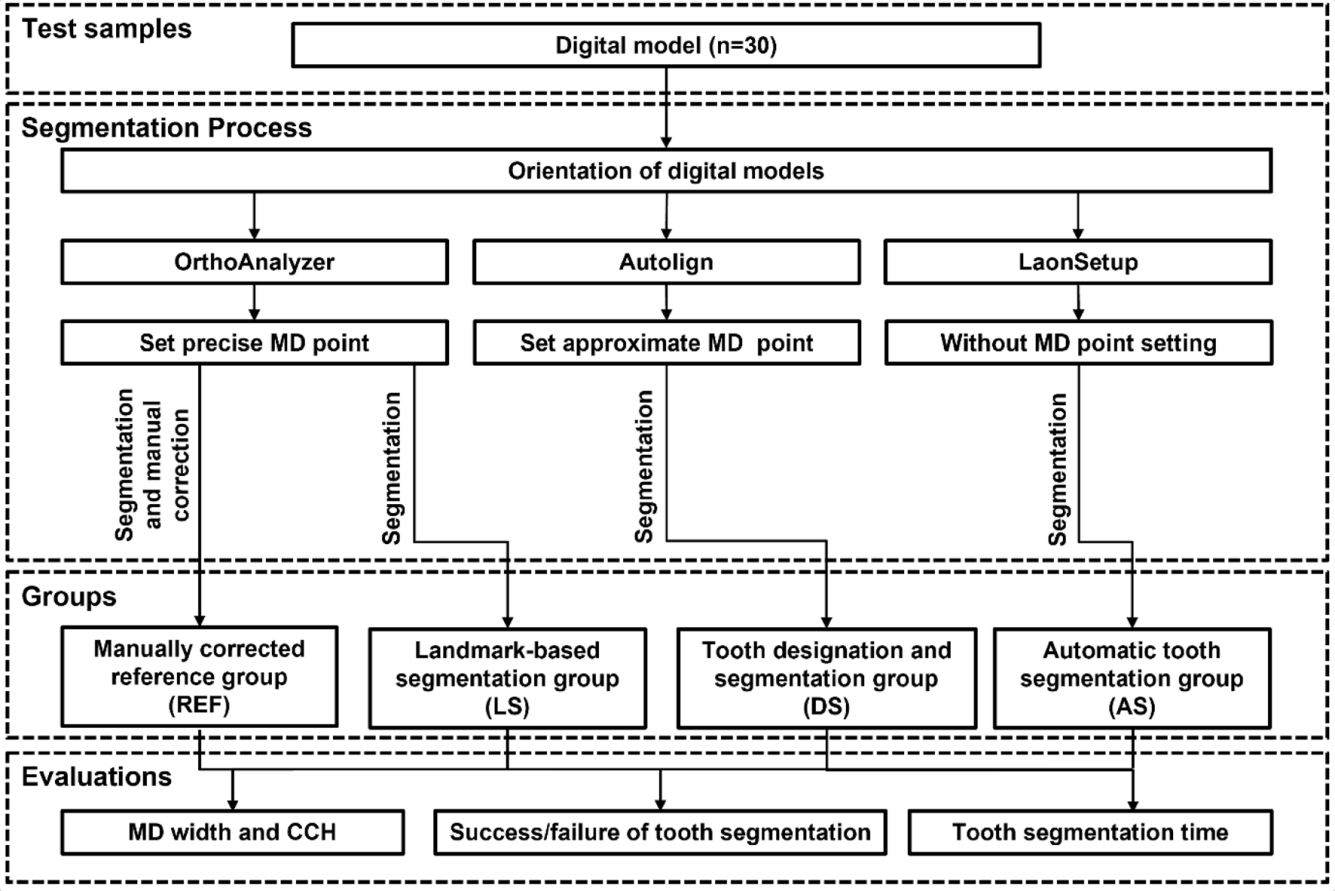
Schematic diagram indicating the study flow. Thirty digital dental models were segmented using three types of software (i.e., OrthoAnalyzer, Autolign, and LaonSetup). The manually corrected segmented tooth was used as the reference group. The size of the segmented teeth such as MD width and CCH, success and failure of tooth segmentation, and tooth segmentation time were evaluated.

**Figure 3:**
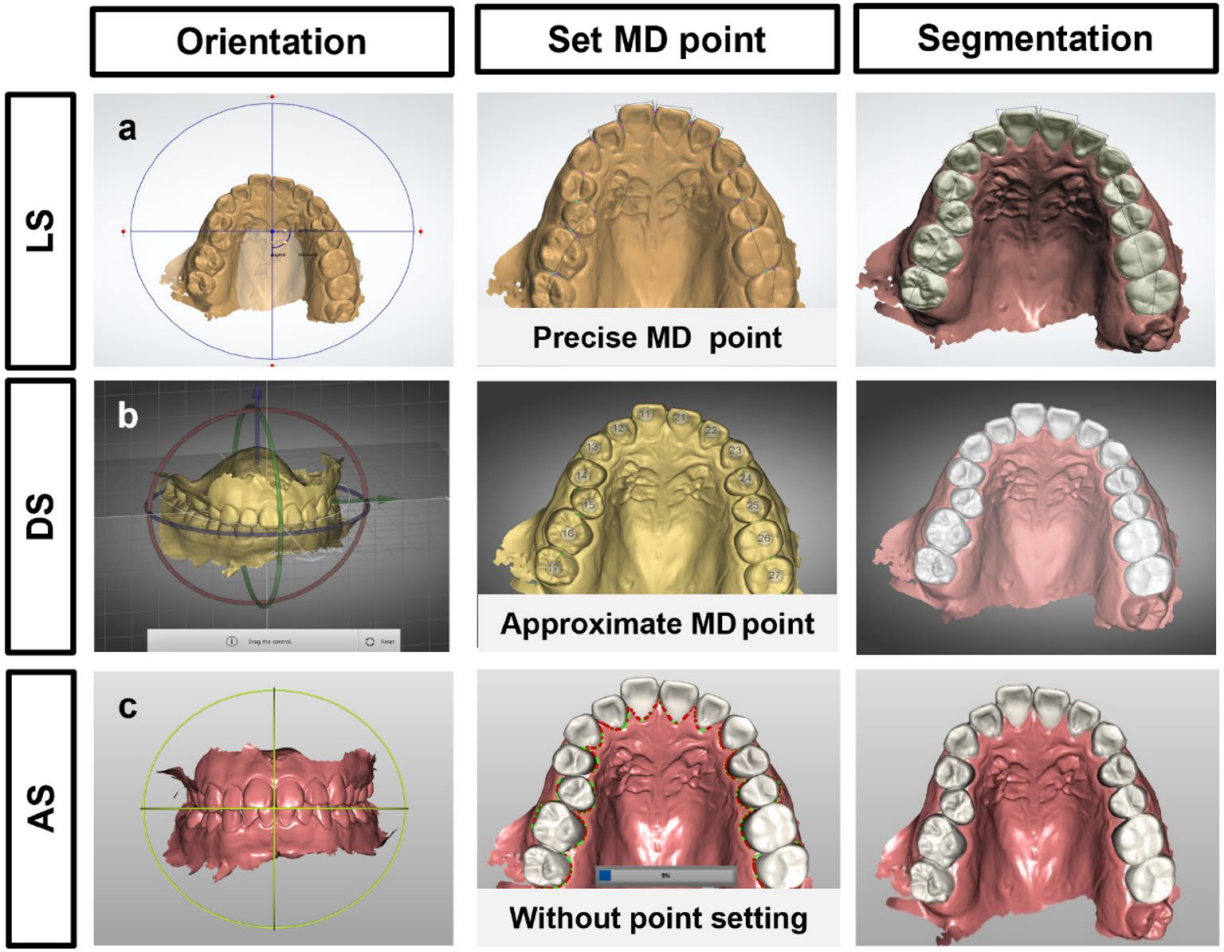
Three different methods of tooth segmentation. The automatic tooth segmentation process has three steps in common: (1) orientation, (2) setting of the mesiodistal (MD) points, and (3) segmentation, but the details differ depending on the software’s algorithm. (**a**) Landmark-based segmentation (LS) using OrthoAnalyzer software: precise MD point setting is required prior to tooth segmentation, and the tooth number and axis are determined. (**b**) Tooth designation and segmentation (DS) using Autolign software: approximate MD point setting is required, and the tooth number is designated. (**c**) Automatic tooth segmentation (AS) using LaonSetup software: fully automatic segmentation based on deep learning does not require MD point setting, and manual intervention is not required.

A schematic diagram for this study is shown in Fig. 2. Thirty identical digital models were segmented by three different methods (Fig. 3). (1) In the landmark-based tooth segmentation method (LS) using OrthoAnalyzer (ver.1.7.1.3, 3shape, Denmark), after orienting the digital model in the virtual coordinate system, the precise MD points of the teeth were set, and the segmentation proceeded. (2) In the tooth designation and segmentation method (DS) using Autolign (ver.1.6.2.1, Diorco, Korea), after orientation of the digital model, the approximate MD points were set, and the tooth segmentation proceeded. (3) In the automatic tooth segmentation method (AS) using LaonSetup (beta version (200722), Laon People, Korea), a deep learning tooth segmentation method based on DGCNN, teeth were segmented without setting the MD points after orientation of the digital model. A manually corrected reference group (REF), containing the spline (interdental and tooth-gingival segmentation lines) corrected by the orthodontic specialist (J.I.) using OrthoAnalyzer, was used as a control group for comparison.

### Measurements

To evaluate the accuracy and efficiency of automatic segmentation using the three types of software, we measured the MD width and CCH of segmented teeth, segmentation time, and success rate. We used a total of 3, 360 teeth, 28 teeth from 30 identical digital models, each segmented in three experimental groups and one control group, for evaluation. Orthodontic specialist (J.I.) measured ten randomly selected digital models twice within a two-week interval to confirm the reliability of the measurements.

#### MD width and CCH of automatically segmented teeth

**Figure 4:**
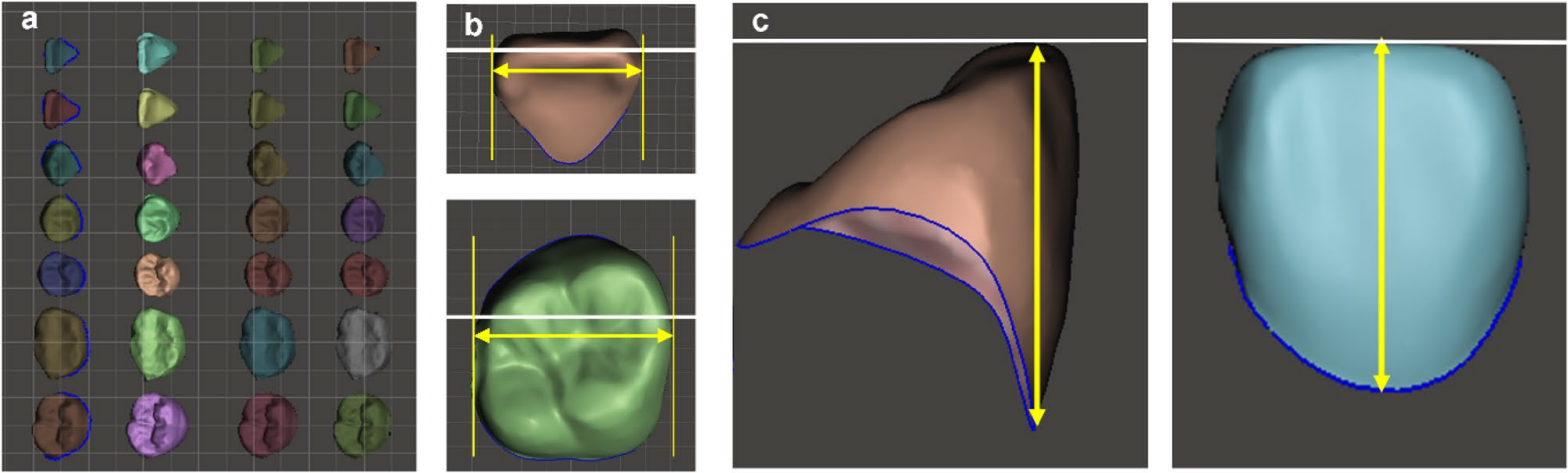
Measurement of the mesiodistal (MD) width and the clinical crown height (CCH) of segmented teeth. (**a**) Reorient the segmented teeth of the LS, DS, AS, and REF groups using Meshmixer software. (**b**) Measurement of the MD width of segmented teeth in REF group. (**c**) Measurement of the CCH of segmented teeth as the distance between the virtual occlusal plane and the lowest point of the gingival margin of the clinical crown.

We extracted the segmented teeth of the three experimental groups (LS, DS, and AS) and the control group (REF) as individual teeth using Meshmixer (ver.11.5.474, Autodesk, USA) and imported into the Geomagic Control X software (3D Systems, USA), where we superimposed the corresponding teeth using the best fit method. Overlapped teeth were imported into Meshmixer and rearranged to measure the MD width and CCH. We established a virtual occlusal plane and positioned the incisal tip and buccal cusp of the overlapping teeth. Then, we adjusted the rotation, angulation, and inclination of the teeth to measure the MD width and CCH. The accuracy of the MD width of the three experimental groups was evaluated using the values output from each program. For comparison, the control group values were measured by Meshmixer. For measuring CCH, the segmented teeth of all groups re-orientated in Meshmixer were loaded into Geomagic Control X software, and the distance between the virtual occlusal plane and the lowest point of the gingival margin of the clinical crown was measured (Fig. 4).

#### Segmentation success rate

**Figure 5:**
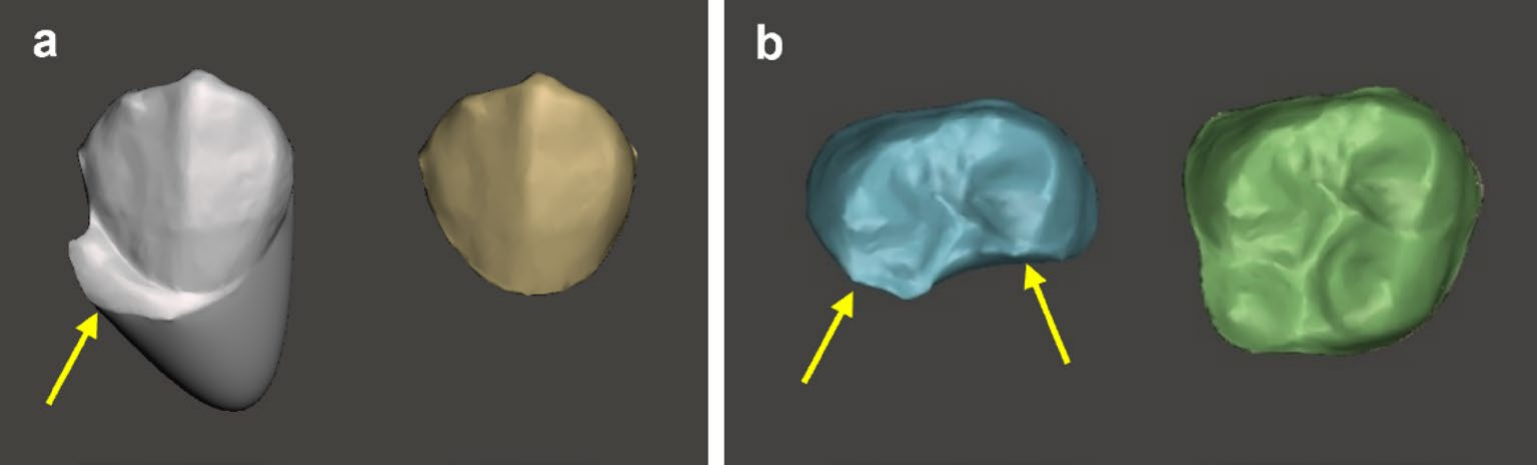
Examples of segmentation failure; an arrow marks the part with segmentation failure. (**a**) Left: segmented tooth beyond the gingival margin; right: reference tooth. (**b**) Left: segmented tooth with partial loss of occlusal surface; right: reference tooth.

The case where the segmented tooth margin did not deviate from ± 25% of the cervical margin of REF group, was set as the success criterion for segmentation. When a segmentation fault was found on one or more occlusal, labial, lingual, or distal surfaces of the most posterior molars, the segmentation of the tooth was considered a failure (Fig. 5). For each experimental group, we determined the segmentation success rate for 30 digital models (i.e. 840 segmented teeth).

#### Segmentation time

We measured the time required for segmentation in seconds after loading the digital model into the software used in each experimental group from the orientation stage to the output of the value of the MD width. In the LS group using OrthoAnalyzer and the DS group using Autolign, it was necessary to mark the mesial and distal points of each tooth (28 teeth) of the digital model after orientation. In the AS group using LaonSetup, the software automatically classified and segmented the teeth.

### Statistical analysis

Data of numerical variables were summarised by their mean (95% confidence interval, 95% CI). The reliability of the measurement of tooth size error was calculated by determining the intraclass correlation coefficient (ICC, two-way random model). Cohen’s kappa determined the reliability of success and failure of tooth segmentation with nominal variables. Differences in variables across groups were compared with Friedman’s test or Cochran’s Q test as appropriate. We used the Shapiro–Wilk test to verify whether the data followed a normal distribution. Independent variables affecting the response (tooth size error) were simultaneously evaluated by a GLMM. GLMM jointly considered the main and the first-order interaction effects. Bonferroni’s test was used for post hoc multiple comparisons. All statistical analyses were performed using SPSS version 26.0 statistical software, and *p* values of less than 0.05 were considered as indicating statistical significance.

## Supplementary Information

Supplementary Figure S1.

## Data Availability

The data underlying this article cannot be shared publicly to protect the privacy of individuals that participated in the study. The data will be shared at reasonable request to the corresponding author.

## Abbreviations

LS: Landmark-based tooth segmentation
DS: Tooth designation and segmentation
AS: Automatic tooth segmentation
MD width: Mesiodistal width
CCH: Clinical crown height
DGCNN: Dynamic graph convolutional neural network
GLMM: Generalised linear mixed model

## References

[1] Macchi A, Carrafiello G, Cacciafesta V, Norcini A (2006). Three-dimensional digital modeling and setup. Am. J. Orthod. Dentofac. Orthop..

[2] Barreto MS, Faber J, Vogel CJ, Araujo TM (2016). Reliability of digital orthodontic setups. Angle Orthod..

[3] Tarraf NE, Ali DM (2018). Present and the future of digital orthodontics. Semin. Orthod..

[4] Nguyen T, Jackson T (2018). 3D technologies for precision in orthodontics. Semin. Orthod..

[5] Kesling HD (1946). Coordinating the predetermined pattern and tooth positioner with conventional treatment. Am. J. Orthod. Oral. Surg..

[6] Kesling HD (1956). The diagnostic setup with consideration of the third dimension. Am. J. Orthod..

[7] Hou D, Capote R, Bayirli B, Chan DCN, Huang G (2020). The effect of digital diagnostic setups on orthodontic treatment planning. Am. J. Orthod. Dentofac. Orthop..

[8] Miller KB (2007). A comparison of treatment impacts between Invisalign aligner and fixed appliance therapy during the first week of treatment. Am. J. Orthod. Dentofac. Orthop..

[9] Fillion D (2011). Lingual straightwire treatment with the Orapix system. J. Clin. Orthod..

[10] Wiechmann D, Rummel V, Thalheim A, Simon J-S, Wiechmann L (2003). Customized brackets and archwires for lingual orthodontic treatment. Am. J. Orthod. Dentofac. Orthop..

[11] González Guzmán, J. F. & Teramoto Ohara, A. Evaluation of three-dimensional printed virtual setups. *Am. J. Orthod. Dentofac. Orthop.* **155**, 288–295. 10.1016/j.ajodo.2018.08.017 (2019). 10.1016/j.ajodo.2018.08.017 30712700

[12] Im J, Cha JY, Lee KJ, Yu HS, Hwang CJ (2014). Comparison of virtual and manual tooth setups with digital and plaster models in extraction cases. Am. J. Orthod. Dentofac. Orthop..

[13] Yuan T, Wang Y, Hou Z, Wang J (2020). Tooth segmentation and gingival tissue deformation framework for 3D orthodontic treatment planning and evaluating. Med. Biol. Eng. Comput..

[14] Kim, T., Cho, Y., Kim, D., Chang, M. & Kim, Y.-J. Tooth segmentation of 3D scan data using generative adversarial networks. *Appl. Sci*. 10.3390/app10020490 (2020)

[15] Tian S (2019). Automatic classification and segmentation of teeth on 3D dental model using hierarchical deep learning networks. IEEE Access.

[16] Qi, C. R., Su, H., Mo, K. & Guibas, L. J. Pointnet: Deep learning on point sets for 3D classification and segmentation. in *Proceeding of the IEEE Computer Society Conference on Computer Visual Pattern Recognition*. 652–660 (2017).

[17] Gao, G. *et al.* 6D object pose regression via supervised learning on point clouds. in *IEEE International Conference on Robotics and Automation (ICRA).* 3643–3649 (2020).

[18] Xu X, Liu C, Zheng Y (2018). 3D tooth segmentation and labeling using deep convolutional neural networks. IEEE Trans. Vis. Comput. Graph..

[19] Zanjani, F. G. *et al.* Deep learning approach to semantic segmentation in 3D point cloud intra-oral scans of teeth. in *Proceedings of the 2nd International Conference on Medical Imaging with Deep Learning (PMLR)*. Vol. 102. 557–571 (2019).

[20] Wang Y (2019). Dynamic graph cnn for learning on point clouds. ACM Trans. Graph. (TOG).

[21] Suhail, Y., Upadhyay, M., Chhibber, A. Kshitiz. Machine learning for the diagnosis of orthodontic extractions: A computational analysis using ensemble learning. *Bioengineering (Basel)* **7**, 55. 10.3390/bioengineering7020055 (2020) 10.3390/bioengineering7020055 PMC7355468 32545428

[22] Choi H-I (2019). Artificial intelligent model with neural network machine learning for the diagnosis of orthognathic surgery. J. Craniofac. Surg..

[23] Xie X, Wang L, Wang A (2010). Artificial neural network modeling for deciding if extractions are necessary prior to orthodontic treatment. Angle orthod..

[24] Lee JH, Yu HJ, Kim MJ, Kim JW, Choi J (2020). Automated cephalometric landmark detection with confidence regions using Bayesian convolutional neural networks. BMC Oral Health.

[25] Kim H (2020). Web-based fully automated cephalometric analysis by deep learning. Comput. Methods Programs Biomed..

[26] Laurenziello M (2017). Determinants of maxillary canine impaction: Retrospective clinical and radiographic study. J. Clin. Exp. Dent..

[27] Kök H, Acilar AM, İzgi MS (2019). Usage and comparison of artificial intelligence algorithms for determination of growth and development by cervical vertebrae stages in orthodontics. Prog. Orthod..

[28] Xu X, Liu C, Zheng Y (2019). 3D tooth segmentation and labeling using deep convolutional neural networks. IEEE Trans. Vis. Comput. Graphics.

[29] Mouritsen, D. A. *Automatic Segmentation of Teeth in Digital Dental Models*. (The University of Alabama at Birmingham, 2013).

[30] Rössl C, Kobbelt L, Seidel H-P (2000). Extraction of feature lines on triangulated surfaces using morphological operators. Proc. AAAI Sympos. Smart Graph..

