# Supplementary material for: Accuracy and efficiency of automatic tooth segmentation in digital dental models using deep learning
Source: Sci Rep. 2022 Jun 8;12:9429. doi: 10.1038/s41598-022-13595-2 (PMC9178028; doi:10.1038/s41598-022-13595-2)
Supplement: Supplementary file 1 — Supplementary Figure S1. [file 41598_2022_13595_MOESM1_ESM.docx]

**Supplementary Figure S1. Overall architecture of the segmentation network model with hyperparameters**


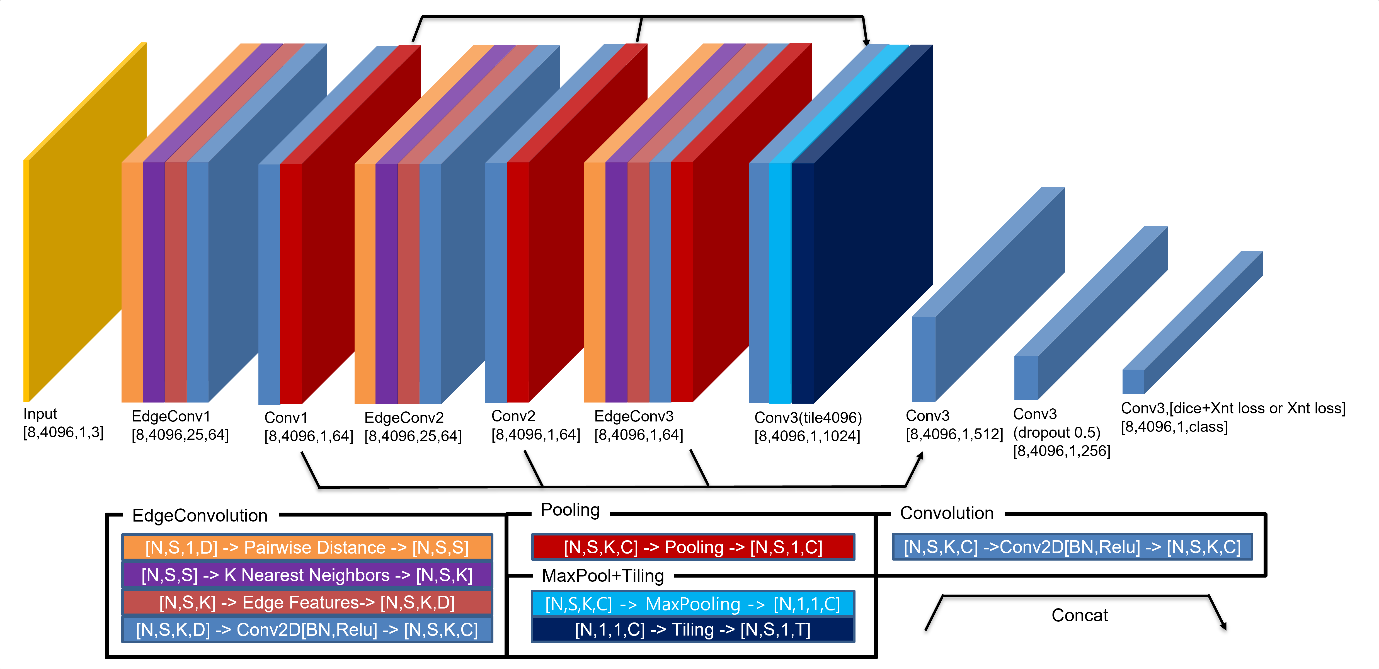


N: Batch size.

S: Number of points.

K: Number of recent points specified for a point in the kNN.

D: Dimension of points input to EdgeConv.

C: Number of filters in the convolution.

T: Tiling

Hyperparameter selection rationale is presented as follows:

N(8): It is better if the batch is large; however, as the edge convolution layer uses a lot of memory, it is maintained small as long as the batch normalization performance does not degrade.

S(4096): Owing to the uniform sampling, the performance is poor when it is too small.

K(25): Using DGCNN defaults.

Batch normalize: Use DGCNN defaults.

Optimizer: Using Adam as DGCNN defaults.

Dropout: 0.5 –the best result from the values between 0.3 and 0.7

activation_function: ReLU(Rectified Linear Unit): use according to existing DGCNN.

Cost (loss function): When performing tooth segmentation, class imbalance occurs because there are fewer tooth vertices than gingiva vertices. To solve this problem, [dice+exnt] was used.
